# Identification and characteristics of muscle growth-related microRNA in the Pacific abalone, *Haliotis discus hannai*

**DOI:** 10.1186/s12864-018-5347-9

**Published:** 2018-12-13

**Authors:** Jianfang Huang, Xuan Luo, Miaoqin Huang, Guangmou Liu, Weiwei You, Caihuan Ke

**Affiliations:** 1 State Key Laboratory of Marine Environmental Science, Xiamen University, Xiamen, 361102 China; 2 College of Ocean and Earth Sciences, Xiamen University, Xiamen, 361102 China; 3 Fujian Collaborative Innovation Center for Exploitation and Utilization of Marine Biological Resources, Xiamen University, Xiamen, 361102 China; 4 National Engineering Research Center of Marine Shellfish, Weihai, 264316 China

**Keywords:** miRNA, Pacific abalone, *Haliotis discus hannai*, Muscle growth, Hdh-miR-1984

## Abstract

**Background:**

The Pacific abalone, *Haliotis discus hannai*, is the most important cultivated abalone in China. Improving abalone muscle growth and increasing the rate of growth are important genetic improvement programs in this industry. MicroRNAs are important small noncoding RNA molecules that regulate post-transcription gene expression. However, no miRNAs have been reported to regulate muscle growth in *H. discus hannai*.

**Results:**

we profiled six small RNA libraries for three large abalone individuals (L_HD group) and three small individuals (S_HD group) using RNA sequencing technology. A total of 205 miRNAs, including 200 novel and 5 known miRNAs, were identified. In the L_HD group, 3 miRNAs were up-regulated and 7 were down-regulated compared to the S_HD specimens. Bioinformatics analysis of miRNA target genes revealed that miRNAs participated in the regulation of cellular metabolic processes, the regulation of biological processes, the Wnt signaling pathway, ECM-receptor interaction, and the MAPK signaling pathway, which are associated with regulating growth. Bone morphogenetic protein 7 (BMP7) was verified as a target gene of hdh-miR-1984 by a luciferase reporter assay and we examined the expression pattern in different developmental stages.

**Conclusion:**

This is the first study to demonstrate that miRNAs are related to the muscle growth of *H. discus hannai*. This information could be used to study the mechanisms of abalone muscle growth. These DE-miRNAs may be useful as molecular markers for functional genomics and breeding research in abalone and closely related species.

**Electronic supplementary material:**

The online version of this article (10.1186/s12864-018-5347-9) contains supplementary material, which is available to authorized users.

## Background

The Pacific abalone, *Haliotis discus hannai*, is the most important cultivated abalone in China [1, 2]. The foot muscle is the main edible portion of the abalone. Improving abalone muscle growth and increasing the rate of growth are important genetic improvement programs [3]. A better understanding of the molecular mechanisms of muscle growth can provide useful knowledge for programs that aim to improve abalone musculature.

MicroRNAs (miRNAs) are small noncoding RNA molecules (18–22 nt) which regulate post-transcription gene expression by specifically mapping target mRNA 3′ untranslated regions (UTRs) [4, 5]. MicroRNAs have crucial roles in various biological processes, including development [6], sex determination and differentiation [7], apoptosis [8], and immune response [9]. MicroRNAs also play a vital role in regulating muscle growth [10, 11]. For example, miR-133 can regulate skeletal muscle proliferation and differentiation by repressing the serum response factor (SRF) and insulin-like growth factor 1(IGF-1) [12]. Seok et al. reported that miR-155 can repress skeletal muscle differentiation by inhibiting the expression of myocyte enhancer factor 2A (MEF2A) protein [13]. The miR-214 target suppressor of fused (Sufu) regulates the slow muscle phenotype in zebrafish [14]. Pm-miR-133 regulates the expression of RhoA in the pearl oyster *Pinctada martensii* [15]. Based on these studies, the identification of miRNAs in adductor muscle could provide new insight into the regulatory mechanism of abalone muscle growth.

In this study, we investigated the miRNA profiles of *H. discus hannai* muscle using an Illumina HiSeq 2500 platform. Differentially expressed miRNAs (DE-miRNAs) related to muscle growth were identified, and the target genes were forecast. The possible roles of the DE-miRNAs and the target genes are discussed. The dynamic expression pattern of hdh-miR-1984 and the predicted target gene bone morphogenetic protein 7 (BMP7) in different developmental stages were examined by quantitative real-time polymerase chain reactions (qRT-PCR). We verified that BMP7 is a target gene of hdh-miR-1984 using the luciferase activities of report vectors method. These data provide new information on the molecular mechanisms of abalone muscle growth.

## Materials and methods

### Experimental samples

A breeding population of *H. discus hannai* produced pedigreed offspring. All of the mating was conducted at Fuda Aquaculture in Jinjiang, Fujian Province, China. Adductor muscle tissue from different growth stages (1, 4, 7, 10, 12, and 24 months) of *H. discus hannai* were acclimated, immediately snap-frozen in liquid nitrogen, and stored at − 80 °C.

### Small RNA sequencing

Adductor muscle tissues of three smaller individual *H. discus hannai* abalones (“S_HD” group) and three larger individuals (“L_HD” group) were used for the sRNA library. The individuals were collected when they were about 2 years old. The total RNA from the abalone samples was isolated using TRIzol reagent (Invitrogen, Carlsbad, CA, USA). Approximately 3 μg total RNA per sample was used for the small RNA library. We performed the single-end sequencing (50 bp) on an Illumina Hiseq2500 platform at Novogene (Tianjing, China) according to the manufacturer’s protocol.

### Small RNA analysis and annotation

After sequencing, clean reads were obtained by removing reads containing the poly-N, poly A/T/G/C, adapter-contaminated tags and low-quality reads from the raw data. Q20, Q30, and GC-content of the raw data were calculated. Then, the downstream analyses were conducted by choosing a certain range of length from clean reads [16]. The small RNA tags were mapped to a reference sequence by Bowtie [17] and then the mapped small RNA tags were used to look for known miRNA. The miRBase20.0 (ftp://mirbase.org/pub/mirbase/20/) was used as reference. Modified software mirdeep2 [18] and srna-tools-cli (http://srna-tools.cm p.uea.ac.uk) were used to obtain the potential miRNA and draw the secondary structures. The software miREvo [19] and mirdeep2 [18] were integrated to predict novel miRNA.

### Differentially expressed (DE) miRNAs

Differential expression of the two groups was analyzed using the DESeq R package (1.8.3) [20]. The *P*-values were adjusted using the Benjamini and Hochberg method [21]. A q-value < 0.05 was set as the threshold for considering differential expression as significant.

### Functional analysis

The target gene of miRNA was predicted by psRobot_tar in miRanda [22]. GOSeq software [23] and KOBAS 2.0 software [24] were used to annotate the functions of the predicted target genes. The miRNA-mRNA interaction networks of DE-miRNAs and their corresponding target genes were constructed using Cytoscape (http://www.cytoscape.org/).

### qRT-PCR

Reverse transcription of miRNA was carried out using a Mir-X miRNA First-Strand Synthesis Kit (Takara, Dalian, China). Several miRNAs were selected for qRT-PCR using gene-specific primers (Additional file 1) and universal reverse primers and U6 was used as the internal control [15]. The qRT-PCR experiments were conducted on a 7500FAST system (ABI, USA). The PCR amplification was performed in 20 μL reaction mixtures containing the following components: 10 μL FastStart Universal SYBR Green Master (ROX), 1 μL forward and reverse primers (10 μM each), 5 μL of cDNA (100-times diluted), 4 μL distilled water. The cycling parameters used were as follows: 95 °C for 10 min, 40 cycles at 95 °C for 10 s, and 59 °C for 30 s. The fluorescent signal intensities were recorded at the end of each cycle. Relative gene expression levels were quantified using the 2^-∆∆CT^ method [25]. Three independent biological replicates were performed. All of the measurements were made in triplicate.

### Luciferase reporter assay

The partial 3′UTR of abalone BMP7 mRNA was amplified by PCR and inserted into the psiCHECK™-2 Vector (Promega, Madison, USA). The primers used to construct plasmids for the luciferase reporter assay are shown in Additional file 1. Hdh-mir-1984 mimic/inhibitor were co-transfected with BMP7 3′UTR psiCHECK™-2 vector into human embryonic kidney 293 T cells by Lipofectamine LTX and PLUS Reagent (Invitrogen, Carlsbad, CA, USA), respectively. At 48 h post-transfection, all of the cells were harvested and normalized firefly luciferase activities (firefly luciferase activities/Renilla luciferase activities) were obtained using the Dual-Glo® Luciferase Assay System (Promega, Madison, USA).

### Statistical analysis

All of the qRT-PCR data were expressed as mean ± standard deviation (SD). Statistical significance was evaluated using SPSS 19.0 (IBM, USA).

## Results

### Analysis and identification of small RNA

**Fig. 1:**
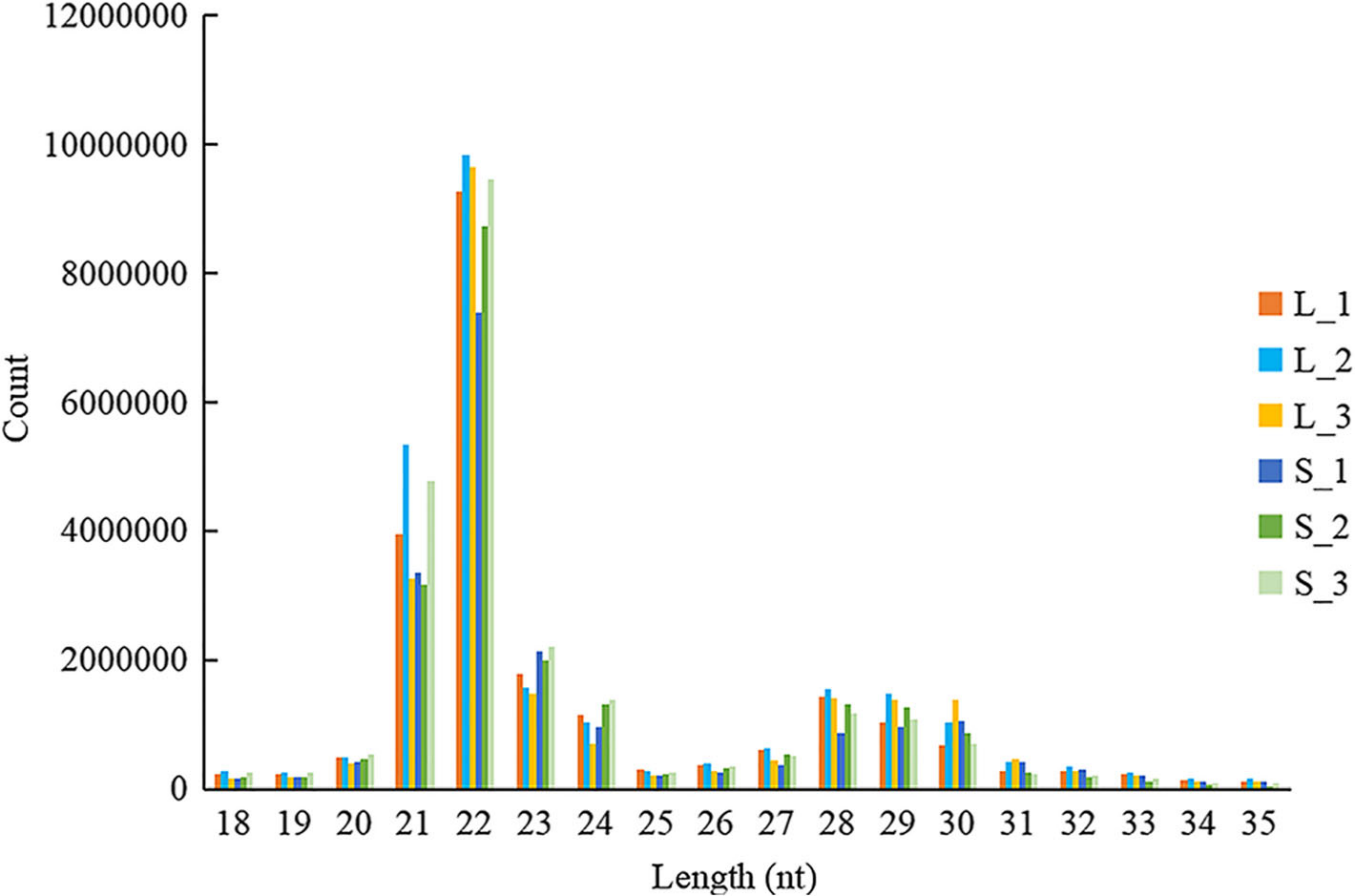
Length comparison of small RNAs from six libraries. Y-axis represents the numbers of small RNA identified in this study. X-axis represents the length of small RNA

Through high-throughput sequencing, 24.92 million raw reads (from 21.41 M to 28.43 M) were obtained (Additional file 2). A total of 24.25 million clean reads (97.33%) remained after removing the N% > 10 reads, the low-quality reads, the adaptor reads, and the poly A/T/G/C reads. A total of 22.27 million sRNAs were annotated and 91.08% of the total sRNAs were successfully mapped to the *H. discus hannai* reference genome (Additional file 3). The majority of the reads ranged from 21 to 23 nt in length and the 22 nt small RNA was the most abundant (Fig. 1). These results confirm the reliability of the small RNA sequencing process used in our study.

To identify the known and novel miRNAs in *H. discus hannai*, small RNA sequences were mapped to the known mature *Haliotis rufescens* miRNAs from the miRBase database. After mapping, 5 known miRNAs and 200 novel miRNAs were identified (Additional file 4). To analyze the conservation of *H. discus hannai* miRNAs, we compared them to all of the species in the miRBase. Only 15 miRNAs were conserved across the different animal species (Additional file 5).

### Differential expression of miRNAs among two groups

**Fig. 2:**
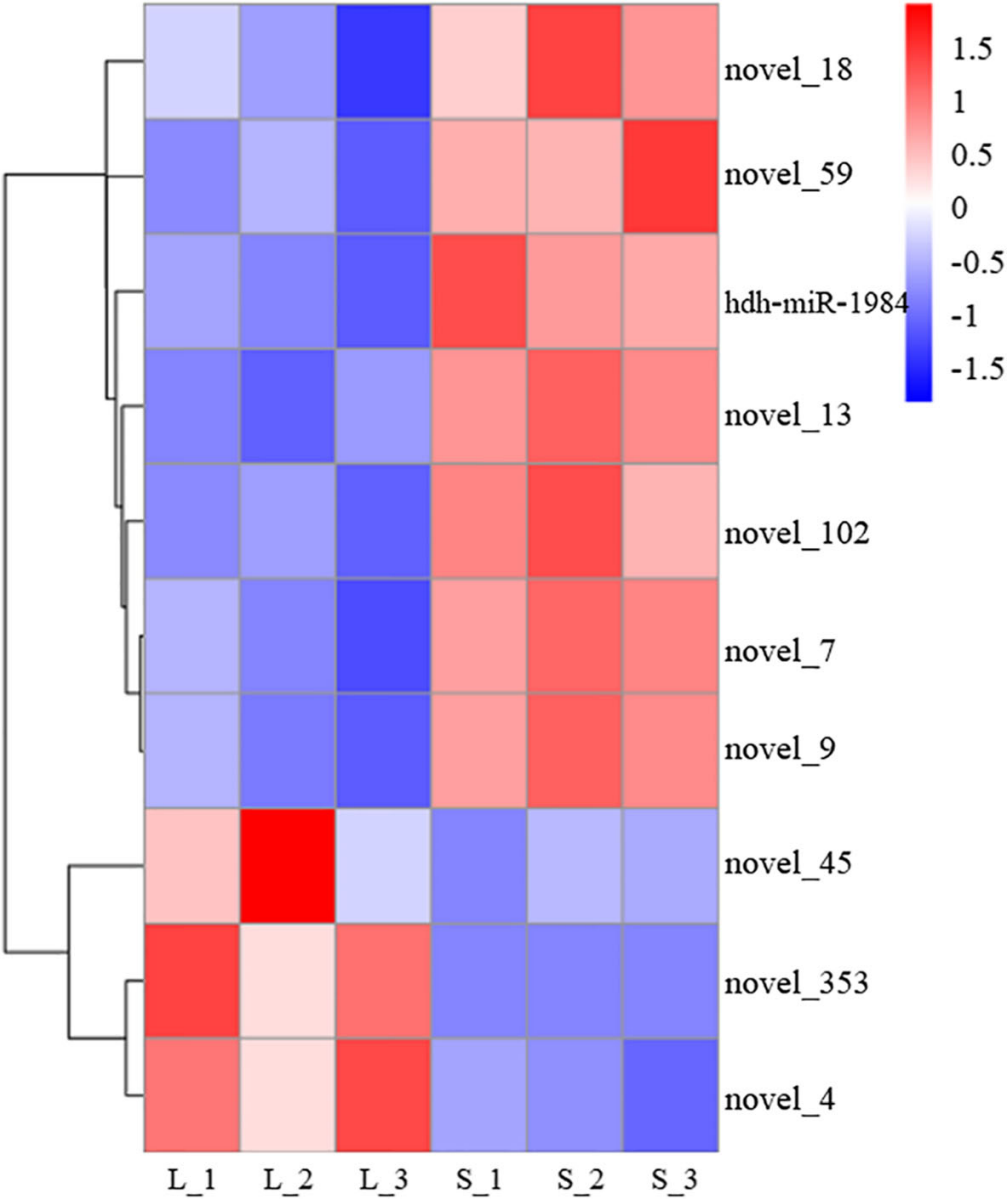
Hierarchical clustering of differentially expressed miRNAs related to the muscle growth. miRNA with a lower expression level is in blue and higher expression in red

We identified 10 miRNAs that were significantly differentially expressed (DE-miRNAs) between the L_HD and S_HD specimens (*P* < 0.05; Table 1). In the L_HD specimens, the novel_353, novel_45, and novel_4 were upregulated compared to the S_HD specimens, while 7 DE-miRNAs were downregulated. Hierarchical clustering analysis (Fig. 2) also suggested that miRNAs were significantly differentially expressed between the two groups.

**Table 1:** The information about significantly different expression of miRNAs between the “L_HD” and “S_HD” groups

sRNA	L_HD_readcount	S_HD_readcount	log_2_ (Fold Change)	*P*-value
novel_353	7.02571687	0	2.4522	5.28E-05
novel_45	549.8169218	127.5556035	1.5764	0.0028014
novel_4	950,955.0449	481,127.1983	0.9533	4.78E-06
novel_13	11,338.62203	18,750.9212	−0.70805	0.00016639
novel_18	4465.909249	8817.185215	−0.91536	0.0026959
novel_9	138,675.92	318,514.975	−1.1718	1.68E-10
novel_7	162,893.4763	402,561.4888	−1.2581	3.14E-08
novel_59	35.18292072	110.4508637	−1.3563	0.0035066
hdh-miR-1984	204,523.75	636,229.4099	−1.5182	1.59E-06
novel_102	1.135605265	9.971214003	−2	0.00038411

### Prediction and annotation of differentially expressed miRNAs targets

**Fig. 3:**
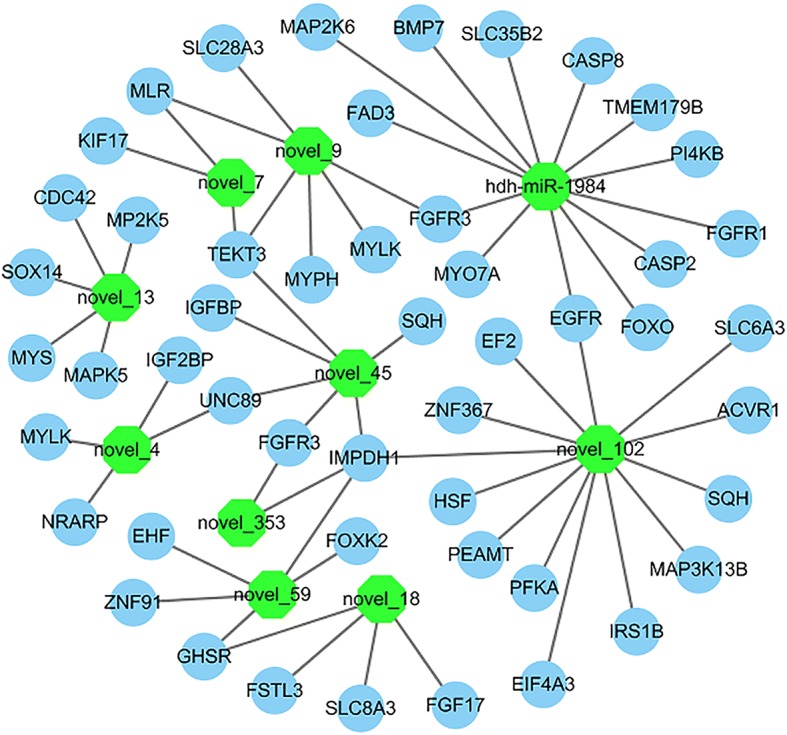
Interaction network of the differentially expressed miRNAs and some of their target genes. The miRNAs are shown in green. Target genes are represented in blue

**Fig. 4:**
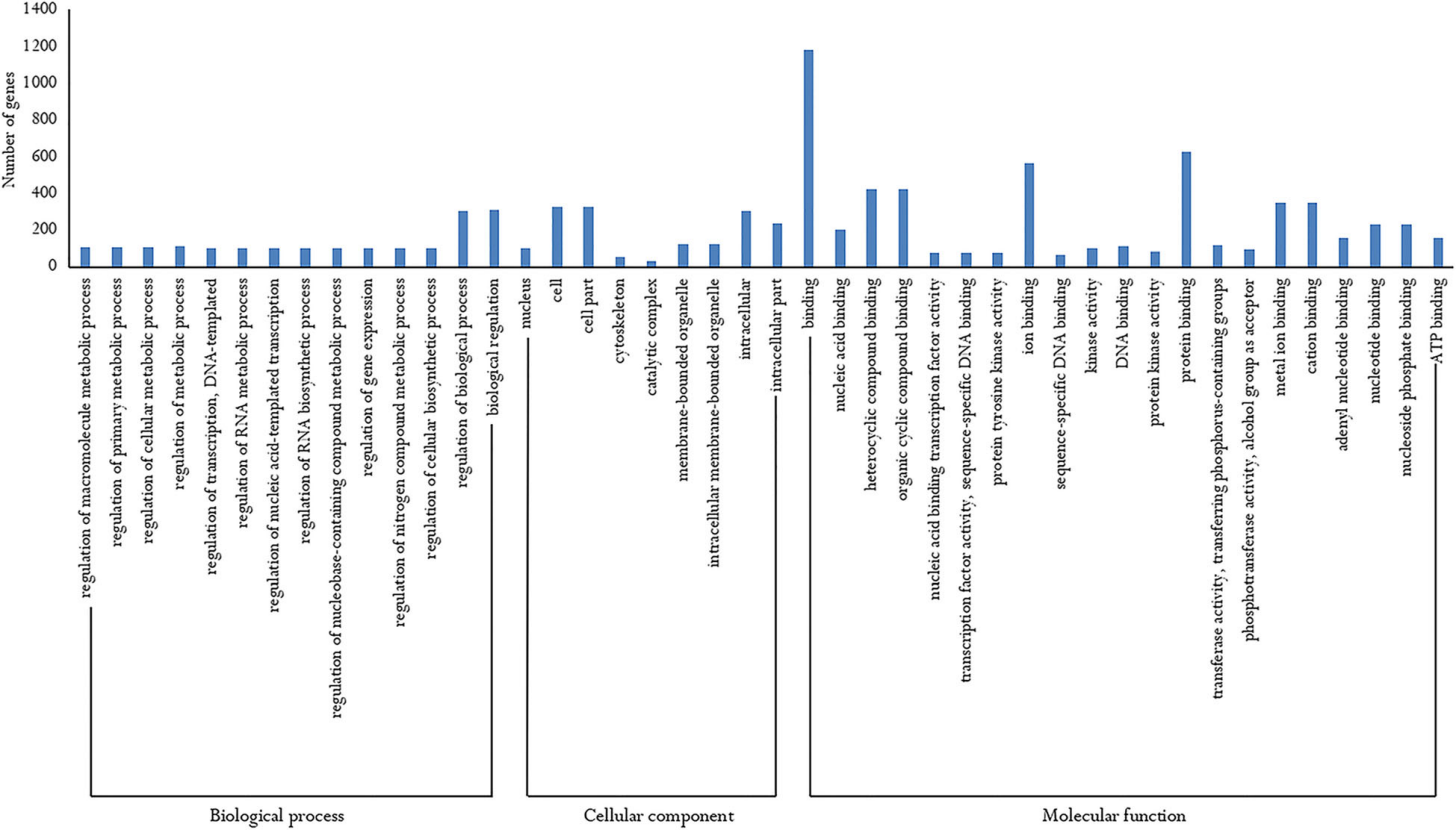
Gene ontology distribution of the target genes for differentially expressed miRNAs

To clarify the molecular functions of the DE-miRNAs in the two groups, we analyzed the target genes of 10 DE-miRNAs. In *H. discus hannai*, 1091 target genes were predicted for 3 up-regulated miRNAs, and 2039 target genes were predicted for 7 down-regulated miRNAs, respectively. Interestingly, some muscle development-related genes were targeted by DE-miRNAs. For example, BMP7, myosin light chain kinase (MYLK), and myosin heavy chain (MYS), were targeted by the hdh-miR-1984, novel_4, and novel_13, indicating that these miRNAs may regulate muscle growth by targeting these genes. Additional file 6 lists some of the potential miRNA target interactions that may play important roles in the muscle growth of *H. discus hannai*. Figure 3 shows a complex network consisting of the DE-miRNAs and some of their target genes. The Gene ontology (GO) distribution of the predicted targets is shown in Fig. 4. These analyses show various biological processes between the “L_HD” group and the “S_HD” group, such as regulation of cellular metabolic process and regulation of biological process. Some targets are categorized as cellular components, including nucleus, cell, cell part, cytoskeleton, catalytic complex, membrane-bounded organelle, and intracellular part. The remaining targets are related to important molecular functions, such as binding, ion binding, and protein binding. A KEGG pathway analysis showed 23 significant pathways (*P*-value < 0.05; Table 2), including the Wnt signaling pathway, MAPK signaling pathway, ECM-receptor interaction, endocrine, and other factor-regulated calcium reabsorption. Several of these terms are primarily involved in growth regulation.

**Table 2:** KEGG pathways of targets of differentially expressed miRNAs between the “L_HD” and “S_HD” *Haliotis discus hannai*

KEGG pathway	Term	*P*-value	Gene Counts	Gene number of Pathway
map 04310	Wnt signaling pathway	1.03E-06	37	156
map 04010	MAPK signaling pathway	4.60E-05	54	293
map 04120	Ubiquitin mediated proteolysis	4.89E-05	48	253
map 04721	Synaptic vesicle cycle	0.000147767	22	88
map 03010	Ribosome	0.000409949	6	307
map 04512	ECM-receptor interaction	0.000409949	18	491
map 04961	Endocrine and other factor-regulated calcium reabsorption	0.000650193	15	54
map 03015	mRNA surveillance pathway	0.001025057	29	144
map 04962	Vasopressin-regulated water reabsorption	0.002088037	17	70
map 04530	Tight junction	0.002088037	37	207
map 05110	Vibrio cholerae infection	0.007001946	23	116
map 04062	Chemokine signaling pathway	0.007001946	29	159
map 04540	Gap junction	0.007001946	29	159
map 04723	Retrograde endocannabinoid signaling	0.007001946	21	103
map 05203	Viral carcinogenesis	0.007001946	42	259
map 00531	Glycosaminoglycan degradation	0.007001946	1	148
map 04912	GnRH signaling pathway	0.007935967	30	169
map 04913	Ovarian steroidogenesis	0.01007854	17	79
map 00564	Glycerophospholipid metabolism	0.034143195	21	114
map 04977	Vitamin digestion and absorption	0.036580765	2	133
map 04390	Hippo signaling pathway	0.036580765	28	168
map 05120	Epithelial cell signaling in Helicobacter pylori infection	0.049867036	19	103

### Validation and expression analysis of identified miRNA

**Fig. 5:**
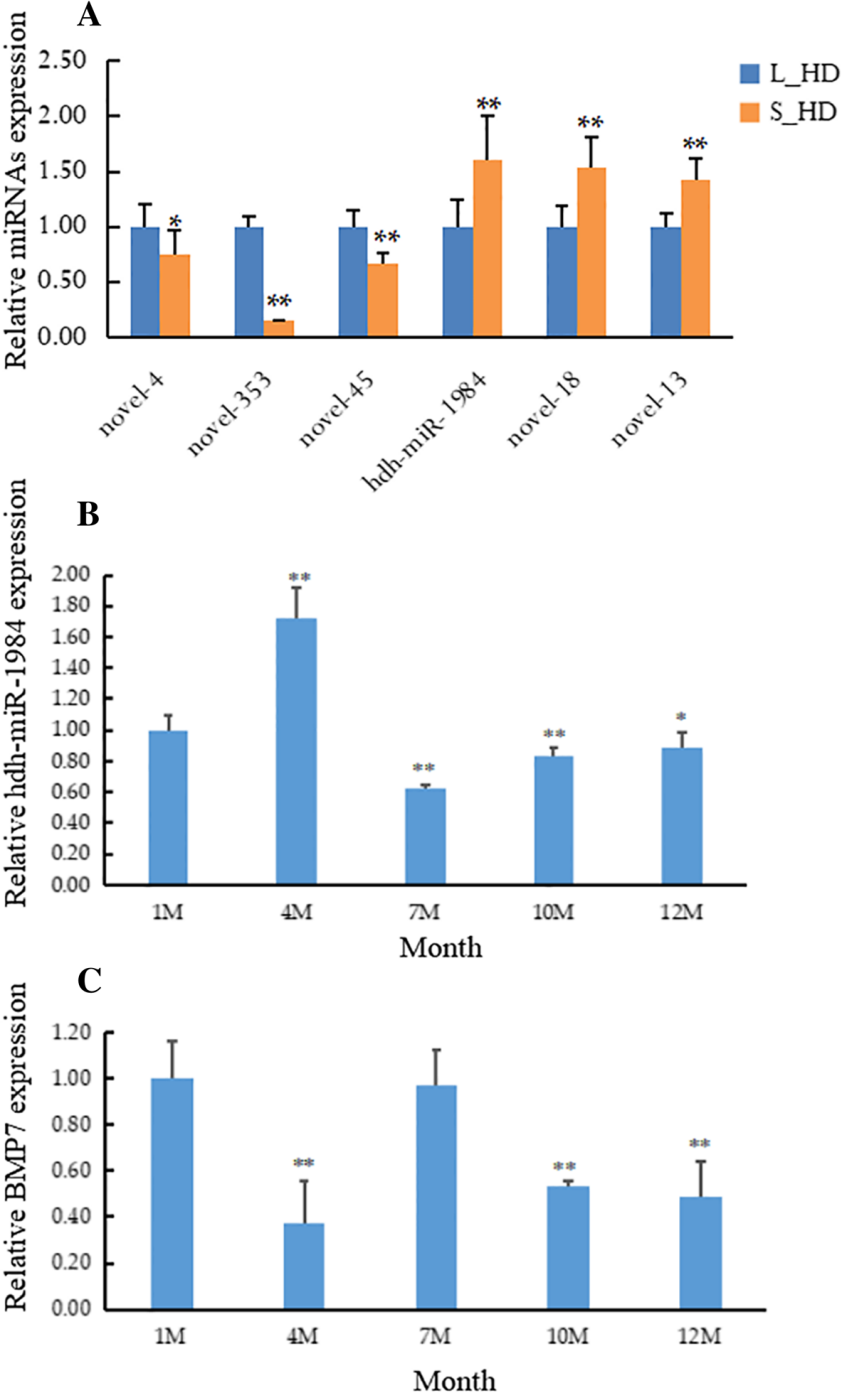
Expression of miRNAs and their target genes quantified with qRT-PCR**.** (**a**) Six differentially expressed miRNAs were examined in the muscle of *Haliotis discus hannai*. The level of (**b**) hdh-miR-1984 and (**c**) BMP7 expression was detected in different age stages of *Haliotis discus hannai*. Values are shown as mean ± SD (*n* = 3). *, *P* < 0.05; **, *P* < 0.01

The differentially expressed miRNA (novel-353, novel-45, novel-4, hdh-miR-1984, novel-18, and novel-13) were validated using qRT-PCR. The expression patterns of these miRNAs were consistent with the small RNA sequencing (Fig. 5a), suggesting high reliability of the small RNA sequencing analysis. We studied the level of hdh-miR-1984 and its putative target gene BMP7 expression in different age stages of *H. discus hannai*. The hdh-miR-1984 and BMP7 had different expression levels at different age stages (Fig. 5b and c) and their expression trends were opposite.

### BMP7 targeted by hdh-miR-1984

**Fig. 6:**
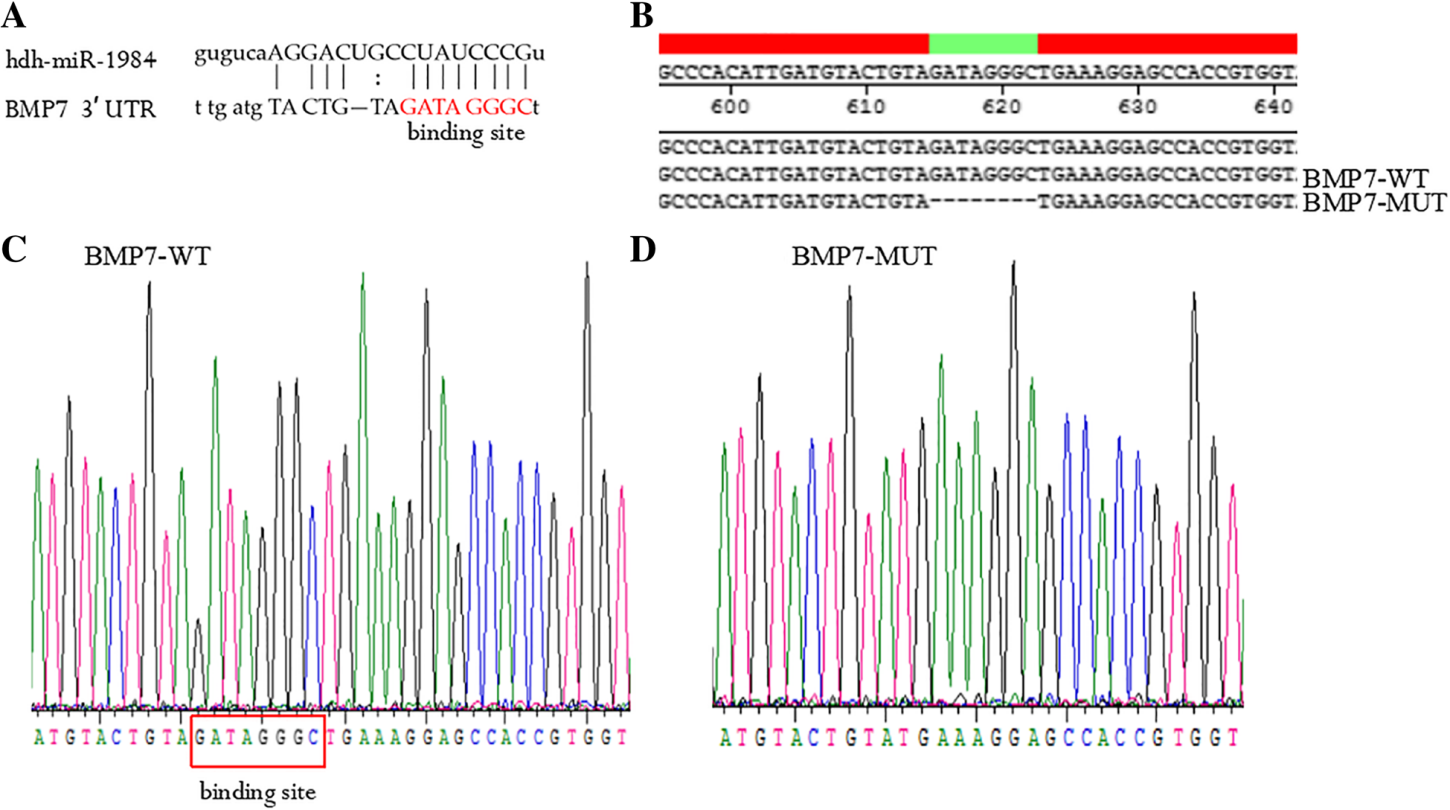
Report of Sequencing results. **a** The seed sequence for hdh-miR-1984 at the 3’UTR of BMP7. **b**, **c**, **d** BMP7-WT vector contains hdh-miR-1984 binding site, and BMP7-MUT vector with mutated seed region of the predicted hdh-miR-1984 sites

**Fig. 7:**
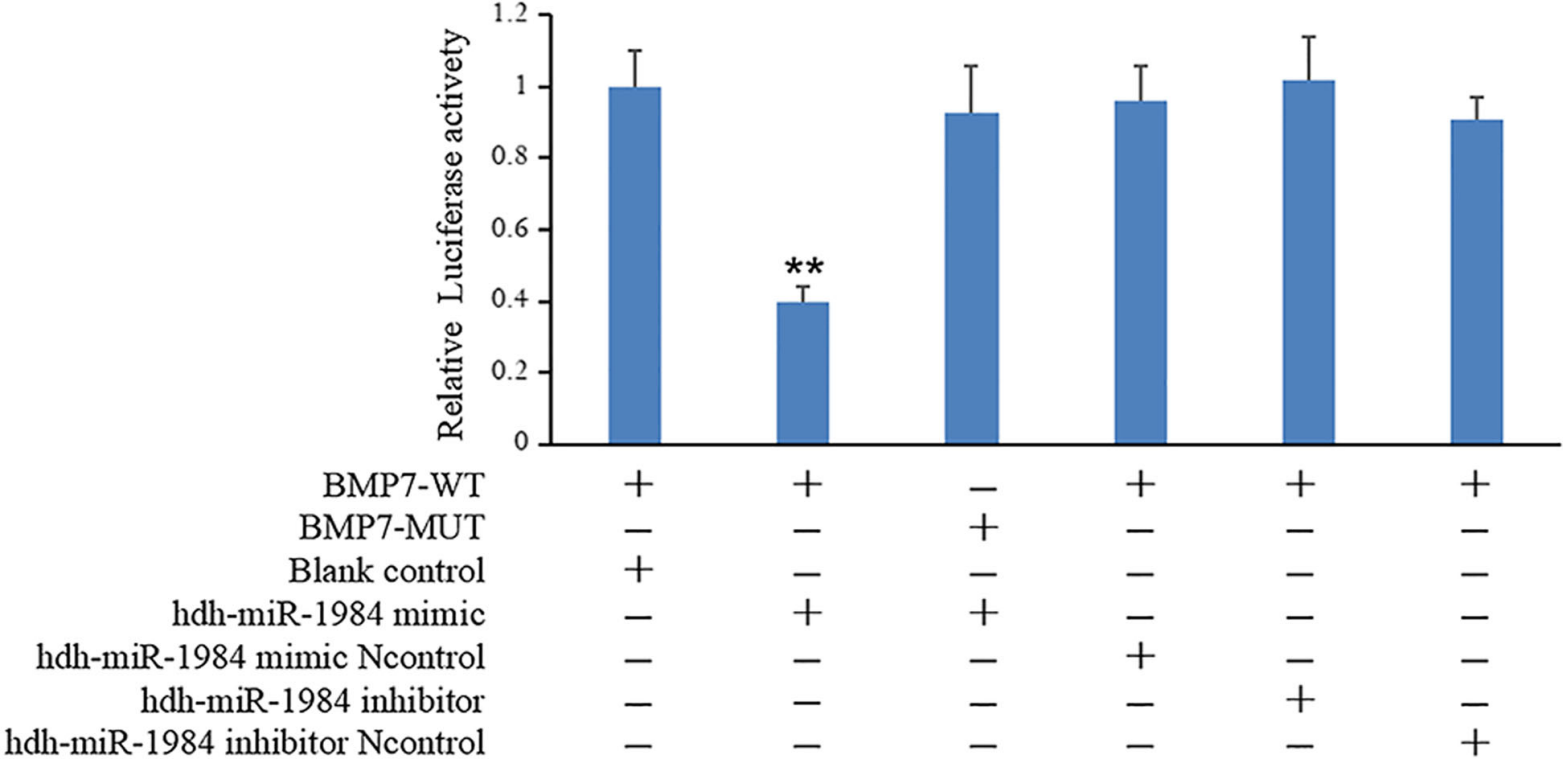
Targeting of BMP7 by hdh-miR-1984. Luciferase activity assay of the wild-type (WT) or mutant (MUT) 3′ UTR of BMP7 using a dual luciferase reporter system in 293 T cell lines following co-transfection with hdh-miR-1984 mimic, mimic Ncontrol, inhibitor, inhibitor Ncontrol, and blank control. In addition, luciferase activities significantly decrease in the BMP7-WT plasmid-transfected 293 T cells following co-transfection with hdh-miR-1984 mimic. Effects are blocked in the mutant plasmids transfected 293 T cells and in the BMP7-WT plasmids transfected 293 T cells following co-transfection with hdh-miR-1984 inhibitor. Data are derived from triplicate transfectants of three independent experiments (** *P* < 0.01)

The putative seed sequences for hdh-miR-1984 at the 3’UTR of BMP7 were indicated based on bioinformatics analysis (Fig. 6a). To confirm that BMP7 is a target of hdh-miR-1984, the wild (BMP7-WT) and mutant (BMP7-MUT) forms of the BMP7 3’-UTR recombinant plasmid vectors were constructed (Fig. 6b, c, d). We then transfected hdh-miR-1984 mimic/mimic Ncontrol/inhibitor/inhibitor Ncontrol and BMP7-WT/BMP7-MUT into the 293 T cells. The luciferase activities of BMP7-WT co-transfection with the hdh-miR-1984 mimic were markedly decreased compared to that with Ncontrol or BMP7-MUT with the hdh-miR-1984 mimic (Fig. 7). Luciferase activities were not repressed in the other co-transfection groups. All of these results indicated that BMP7 is a target of hdh-miR-1984.

## Discussion

In many eukaryotes, miRNA plays a vital role in biological processes [26]. High-throughput sequencing technologies have enabled large-scale studies on miRNA transcriptome profiles in various mollusks, such as *Littorina littorea* [27]*, Crassostrea gigas* [28], *Pinctada martensii* [29], and *Haliotis midae* [30]. In this study, six small RNA libraries were sequenced to identify the miRNAs in the muscle of *H. discus hannai*. Length distribution analysis showed that the most abundant reads were 22 nt, which was consistent with other marine animals [16, 31] and demonstrating that the reads from *H. discus hannai* were reliable and suitable for further analysis. In total, 205 miRNAs were identified in *H. discus hannai* muscle, among which, 15 miRNAs were conserved and 190 were novel among various animal species. Among all the miRNAs, hdh-miR-1984 and hdh-mir-1986 are the only miRNAs which appear to be mollusk-specific. These results suggest that there were mollusk-specific miRNAs in abalone and those 205 miRNAs expressed in *H. discus hannai* muscle might be involved in the modulation of muscle growth. However, the abalones displayed significantly different growth rate in the same cage, while the body weight for the L_HD individuals could be 5 times than the S_HD individuals. The reasons for the differences could be internal genetic factors but also external environmental factors. Therefore, samples from different culture environment will be collected and further analyzed to reveal the molecular mechanism of abalone growth in future.

The miRNAs and epigenetic modifications are major components of the myogenic regulatory network [32]. However, the information of myogenic related miRNAs in abalone remains unknown. To study the probable function of miRNA in abalone muscle growth, the expression profile of miRNAs in *H. discus hannai* muscle was analyzed between the L_HD and S_HD abalones. In the L_HD abalones, 7 miRNAs were down-regulated and 3 miRNAs were up-regulated compared to the S_HD specimens. These results indicate that the 10 DE-miRNAs may be related to growth. Identification of targets can deepen our understanding of the biological roles of miRNA [26]. In this study, the target genes of the 10 muscle growth-related miRNAs were predicted and annotated. The complex network as shown in Fig. 3 suggest that there is a many-to-many interaction relationship between miRNAs and their target genes. The GO terms were associated with many biological functions, such as regulation of gene expression, intracellular membrane-bounded organelle, regulation of cellular biosynthetic process, and binding. The KEGG pathways, including ECM-receptor interaction, Wnt, and the MAPK signaling pathway, were primarily involved in muscle growth regulation. The Wnt signaling pathway plays a important function in the regulation of muscle development [33]. The MAPK signaling pathway is a positive regulator in muscle development [34, 35]. All of the results indicate that these DE-miRNAs might influence the regulation of muscle growth in *H. discus hannai* by affecting target genes.

Muscle development is a complex system regulated by a cascade of factors containing miRNAs. The miRNAs negatively regulate gene expression by binding to the 3′UTR of the target gene in a sequence-specific manner at the posttranscriptional level [36]. The miR-378 downregulates MyoR, an MyoD inhibitor, by recognizing its 3′UTR [37]. The miR-155 inhibited muscle differentiation by repressing expression of the MEF2A [13]. BMPs, as members of the transforming growth factor β (TGF-β) super family, are usually considered potent inhibitors of muscle differentiation [38–41]. BMP7 stimulates Pax-3 expression in low concentration, but high concentration BMP7 induces muscle loss [42]. The miR-378 could suppress BMP2 by targeting its 3′UTR to regulate myogenesis [43]. In this study, hdh-miR-1984 was downregulated in the L_HD abalones compared to the S_HD individuals. Many muscle growth-related target genes were predicted containing the fibroblast growth factor receptor 3 (FGFR3), Myosin-VIIa, epidermal growth factor receptor (EGFR), and BMP7. The hdh-miR-1984 and BMP7 had different expression levels during different age stages. Interestingly, the expressions of hdh-miR-1984 and BMP7 varied in an inverse manner which suggest BMP7 may be a target gene of the hdh-miR-1984. Furthermore, the dual-luciferase reporter assay results indicate that hdh-miR-1984 directly recognized the BMP7 3′ UTR in abalone. All of these imply that hdh-miR-1984 may be a vital muscle growth-related miRNA that regulates muscle growth by targeting BMP7. However, the function of the other detected miRNAs needs further study.

The miRNA have emerged as important roles in the regulation of gene expression. These DE-miRNAs will further be used as molecular markers to screen for fast-growing strains of abalone. In summary, this research is the first analysis of miRNAs in *H. discus hannai* using the Illumina HiSeq sequencing platform. A total of 205 miRNAs were identified, among which, 10 DE-miRNAs were closely associated with muscle growth. In addition, we verified the BMP7 is a target gene of hdh-miR-1984 and examined their dynamic expression pattern in different developmental stages. Taken together, our findings provide useful information for understanding the epigenetic regulation of muscle development and also help to reveal the mechanisms of abalone muscle growth. These findings will further be used to improve artificial selection efficiency and contribute to the genetic improvements of the abalone aquaculture.

## Additional files

Additional file 1: Primers used for PCR and qRT-PCR. (XLSX 10 kb)

Additional file 2: Small RNA sequencing Data. (XLSX 11 kb)

Additional file 3: Genomic location information of Small RNA. (XLSX 10 kb)

Additional file 4: The information of known miRNAs and novel miRNAs. (XLSX 18 kb)

Additional file 5: The conserved miRNAs across the different animal species in the miRBase. (XLSX 10 kb)

Additional file 6: The potential miRNA-target interactions which may play a vital role in muscle growth of the *H. discus hannai*. (XLSX 13 kb)

## Abbreviations

3′UTRs: 3′ untranslated regions
BMP7: bone morphogenetic protein 7
DE: differentially expressed
EGFR: epidermal growth factor receptor.
FGFR3: fibroblast growth factor receptor 3
*H. discus hannai*: *Haliotis discus hannai*
IGF-1: insulin-like growth factor 1
MEF2A: myocyte enhancer factor 2A
miRNAs: MicroRNAs
MYLK: myosin light chain kinase
MYS: myosin heavy chain
qRT-PCR: quantitative real-time polymerase chain reactions
SRF: serum response factor
TGF-β: transforming growth factor β
